# Effects of Parasitism on the Population Growth of *Toumeyella martinezae* (Coccidae) in the Presence of Its Mutualistic Ant *Liometopum apiculatum* (Formicidae) in an Arid Region of Central Mexico

**DOI:** 10.3390/insects16101002

**Published:** 2025-09-26

**Authors:** Alicia Callejas-Chavero, Carlos Fabián Vargas-Mendoza, Humberto González-Villa, Arturo Flores-Martínez

**Affiliations:** 1 Laboratorio de Ecología Vegetal, Escuela Nacional de Ciencias Biológicas, Instituto Politécnico Nacional, Mexico City 11340, Mexico; alicallejas@hotmail.com (A.C.-C.); humberto_biol@hotmail.com (H.G.-V.); 2 Laboratorio de Variación Biológica y Evolución, Escuela Nacional de Ciencias Biológicas, Instituto Politécnico Nacional, Mexico City 11340, Mexico; fvargas@ipn.mx

**Keywords:** biological control, cactus pest, soft scale, population growth, parasitoid

## Abstract

The soft scale *Toumeyella martinezae* infests the arborescent cactus *Myrtillocactus geometrizans*. This scale is, in turn, parasitized by the wasp *Mexidalgus toumeyellus* and forms a mutualistic relationship with the ant *Liometopum apiculatum*. This study assessed how ant and/or parasitoid presence influenced parasitism rates and the population growth of the scale insect. Experimental treatments included scale populations with ant access (control) or ant exclusion, and parasitoid exclusion with ant access. Scale population growth rates were estimated using Lefkovitch projection matrices, built based on the individual monitoring of approximately 5400 scales. The average parasitism rate was higher in the “with ants” treatment (18.66%) than under ant exclusion (5.42%). In the absence of parasitoids, the scale population growth rate (λ = 1.532) was 8% higher than in the control treatment (λ = 1.423). Population growth was negative (λ = 0.636) when ants were excluded. These results indicate that interaction with the mutualistic ant is the primary factor sustaining a positive scale population growth. In contrast, the impact of the parasitoid alone is insufficient for effectively controlling the soft scale pest.

## Simple Summary

Pests in agroecosystems reduce crop productivity. Parasitoid wasps are effective biological control agents for agricultural pests; however, their role in natural ecosystems remains uncertain. In the arid regions of central Mexico, the soft scale insect *Toumeyella martinezae* infests the cactus *Myrtillocactus geometrizans*, the fruits of which are highly valued locally. This scale insect maintains a mutualistic relationship with ants and is targeted by a parasitoid wasp. We evaluated the effect of the ant presence on the parasitoid’s effectiveness in controlling the scale insect. Using exclusion experiments, we measured parasitism rates and scale population growth under varying combinations of ant and parasitoid presence. Parasitoids reduced scale population growth, but not sufficiently for effective pest control. Ant presence was more important in sustaining scale populations with positive growth rates. Effective management of this pest should therefore focus on reducing the ants’ protective role, with parasitoids used as a complementary strategy.

## 1. Introduction

Parasitoids are a diverse group of insects distributed across multiple orders. Approximately 90% of known parasitoid species belong to the orders Hymenoptera and Diptera, while the remaining 10% are found within the orders Coleoptera, Strepsiptera, and Lepidoptera [1,2,3,4].

Parasitoid wasps develop and feed inside or on other arthropods during their larval stage, killing them at the end of the process [5]. This group is very common in nature, with more than 65,000 species described so far [6], and estimates approaching one million species [7]. Parasitoid–host interactions may thus be the most common ecological interactions in terrestrial ecosystems [8].

The use of natural enemies has emerged as a highly effective method of pest management due to their ability to self-propagate, self-perpetuate, and actively seek out hosts [9]. Parasitoids are frequently used in biological control because they maintain a stable ecosystem balance by killing hosts [10]; exhibit high host specificity [6]; efficiently locate hosts even at low densities [11]; synchronize life cycles with those of their hosts [12]; respond to variations in host density and can survive periods of host scarcity [13]; and adapt to environmental changes and exhibit high reproductive success [6].

While searching for hosts, parasitoids use a variety of cues that may originate from plants, hosts, mutualists, or their interactions [14]. These signals include chemical (e.g., odors), physical (e.g., texture, movement), or visual (e.g., colors and shapes) cues [13].

Most studies on plant–herbivore–parasitoid interactions have been conducted under controlled conditions (greenhouses or monocultures), demonstrating a high effectiveness of parasitoids at controlling herbivore populations [13,15]. However, in diverse agroecosystems and natural habitats, complex interactions with other species can reduce their effectiveness [16]. This complexity makes evaluating the impact of parasitoids as biological control agents challenging. For example, parasitism rates observed in natural systems are substantially lower than those observed in monocultures, and may fail to curb pest population growth [17,18].

Plants are important resources for a wide variety of animals, including sap-sucking insects such as scale insects and aphids. These insects often excrete a sugar-rich resource, commonly called honeydew, that is consumed by ants and other insects. The relationship between ants and honeydew-producing insects (Hemiptera) is common in nature and has been widely studied; it can range from mutualism (e.g., [19]) to antagonism (e.g., [20,21]. Mutualistic ants feed on the honeydew produced by insects and, in turn, benefit them by removing excess exudate and preventing suffocation or fungal contamination; increasing host growth rates and shortening development time; and relocating host insects to more suitable feeding and settling sites [22,23].

Ants also defend scale insects against predators and parasitoids [24,25,26,27], either by aggressive attack or by their mere presence. However, this protection is often incomplete and cannot always provide their mutualists with an enemy-free space [28,29,30,31,32]. Moreover, the care provided by ants can improve the host condition and increase its population size, making them more attractive to parasitoids, potentially inducing high parasitism rates despite ant attendance [33,34].

Our team has been conducting extensive research on the arborescent cactus *Myrtillocactus geometrizans* (Mart. ex Pfeiff.) Console 1897 (Cactaceae) (locally known as garambullo) and its interactions with insects in agricultural and natural ecosystems in Mexico. This cactus species is endemic to Mexico and highly prized for its flowers and fruits. Over the last decade, we have observed that infestation by the soft scale *Toumeyella martinezae* (Kondo and González, 2014) (Hemiptera: Coccidae) has been linked to mortality of adult cacti within months of infestation [35]. This has raised concerns among local people and has prompted the need to explore alternative solutions to address the proliferation of this pest.

Our research has revealed that *Toumeyella martinezae* coexists and competes with another scale, *Opuntiaspis philococcus* (Cockerell, 1893) (Hemiptera: Diaspididae) (armed scale), and maintains a mutualistic interaction with the ant *Liometopum apiculatum* (Mayr, 1870) (Hymenoptera: Formicidae), while suffering parasitism by the wasp *Mexidalgus toumeyellus* (Myartseva, 2014) (Hymenoptera: Chalcidoidea) [35,36].

In this study, we evaluate the potential of *M. toumeyellus* as a biological control agent against *T. martinezae* and examine how the presence of *L. apiculatum* influences this interaction. We evaluated parasitism rates, analyzed the parasitoid’s impact on scale demography, and tested the hypothesis that ant attendance reduces parasitism, thereby promoting scale population growth.

## 2. Methods

### 2.1. Study Site and System

This study was conducted in a semi-arid region of central Mexico (20°22′47.2″ N–99°36′23.9″ W). The area features xeric scrubland subject to disturbance by grazing, firewood collection, and agriculture. Dominant species include legumes (*Mimosa, Vachellia*, and *Eysenhardtia*) and cacti (*Myrtillocactus geometrizans*, *Pachycereus marginatus*, *Opuntia* spp.).

The soft scale of the garambullo, *Toumeyella martinezae*, is a sessile phytophagous with a convex, oval body that measures 4.2 to 7.2 mm long and 3.1 to 5.7 mm wide. It is colored pale green with a dark green marginal band. Its life cycle comprises the egg, four nymphal instars defined by body length, and the adult stage. The egg measures approximately 179 µm. The nymphal instars measure 0.6–0.7 mm (first), 0.71–1.21 mm (second), 1.22–3.0 mm (third), and 3.1–5.0 mm (fourth). Adults measure 5.1–7.82 mm [36,37]. Body length is strongly correlated with the development of the anal plate and spiracular setae, both key morphological traits for species identification. First-stage nymphs are mobile for a few hours and are commonly referred to as walkers.

This species is bivoltine, and has been reported only in the locality of Huichapan, Hidalgo, central Mexico, where it is associated exclusively with *M. geometrizans* and coexists with the armored scale *Opuntiaspis philococcus*.

This scale forms a mutualistic relationship with the ant *L. apiculatum*, which tends the scales day and night, feeding on the honeydew they produce, grooming, relocating them to sites suitable for settlement and development, and deterring potential predators and parasitoids [36].

*Liometopum apiculatum*, locally known as the “escamolera ant”, has been highly valued since pre-Hispanic times for the nutritional and economic value of its larvae and pupae. They are the main ingredient of several recipes in traditional Mexican cuisine. The distribution of *L. apiculatum* extends from the southern United States to central Mexico. It inhabits mainly oak forests, xerophytic scrublands, and grasslands at altitudes ranging from 1870 to 2900 m above sea level. In the field, these ants feed mainly on honeydew obtained from various hemipterans, as well as floral nectar or extrafloral nectaries, pollen, oils, and a wide variety of arthropod prey [36].

In the study area, *L. apiculatum* is part of a complex system of interactions involving ants (e.g., *Camponotus*, *Crematogaster*), the armored scale insect (*O. philococcus*), membracids, several species of cacti with extrafloral nectaries (e.g., *Opuntia imbricata, Pachycereus, marginatus, Ferocactus latispinus*), and the Peruvian pepper tree *(Schinus molle*), among other interacting species.

*Mexidalgus toumeyellus* (Hymenoptera: Aphelinidae) is a recently described [38] solitary koinobiont parasitoid (i.e., it allows the host to continue developing while the parasitoid develops by feeding on it) of *T. martinezae* second- and third-instar scales and produces multiple generations per year. To identify the species studied, six to ten specimens per species were collected in the field, and one to three per developmental stage in the case of scales. The organisms were preserved in 70% alcohol until they were examined under a microscope. The species were identified by specialists of each group.

### 2.2. Parasitism Rate

**Figure 1:**
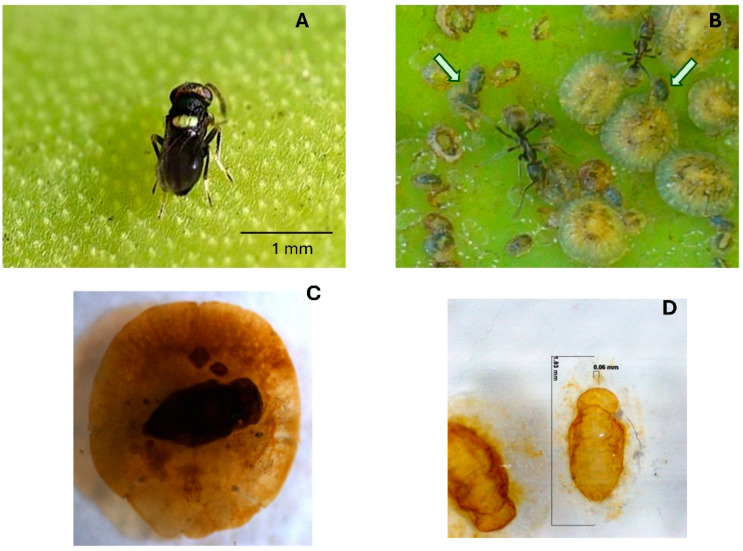
(**A**) *Mexidalgus toumeyellus*. (**B**) Scales tended by *Liometopum apiculatum*; arrows indicate scales infested by the parasitoid. (**C**) *M. toumeyellus* developing within a scale. (**D**) Pupa of *M. toumeyellus*. Photographs by Jesús Luna.

To quantify parasitism, we monitored scale populations on nine *M. geometrizans* individuals over six months. Two branches were selected from each host plant (≥1.5 m tall, hosting scales and ants): one control (unmanipulated branch) and one where ants were excluded with a ring of natural petroleum jelly at the base. Natural petroleum jelly is an odorless, colorless paste that acts as a physical barrier, preventing ants from passing through. It can be applied directly to stems and remains in place for several months. According to our observations in previous studies, it does not alter parasitoids’ behavior. On each branch, a 12 × 6 cm (72 cm^2^) square containing at least 500 scales was delineated. Digital photographs were taken biweekly from a 15 cm distance and including a size reference scale. The photographs were reviewed using MotiCImages 2000 V1.3 [39]. We recorded the total number of scales and their developmental stage, as well as the number of newly parasitized individuals (identified by internal parasite-induced color change, Figure 1) on each photograph.

Parasitism rates were compared between treatments using a Generalized Lineal Mixed Model (GLMM) with a binomial error distribution and logit link function, including ant presence/exclusion as a fixed-effects factor and individual plant as a randomized effect factor. The GLMM was used to account for the variation among plants and reduce the problem of overdispersion, which is a common problem in simple Generalized Linear Models [40,41]. Analyses were performed in JASP 0.95 [42].

### 2.3. Demographic Effects of Parasitoids and Ants on Scale Populations

To assess how ants and parasitoids influence scale population growth, we set up three different treatments on nine *garambullo* individuals: the two treatments described in the preceding section plus a third treatment in which ants were allowed to enter but parasitoids were excluded by covering the branches with a fine mesh that allowed ant access but blocked flying insects. The light-colored mesh used to exclude parasitoids allows light and air to pass through, preventing significant changes in the microclimatic conditions on the branches where the scales are established. As the scales are sessile, the mesh does not affect their mobility. The mesh does not come into contact with the branches’ tissue, allowing ants to move freely over the branch surfaces and allowing access for the scales. As the mesh only acts as a physical barrier, we do not consider it to affect parasitoid biology.

This third treatment showed the behavior of scales tended by ants in the absence of parasitoids, allowing the parasitoid’s net effect on the scale population growth rate to be assessed under natural conditions.

Population growth rates (λ) were estimated with Lefkovitch projection matrices [43]. Six life-stage classes were considered: egg, walker (E1), three immobile instars (E2–E4), and adults. Between-stage transition and persistence probabilities were calculated by individually monitoring between 1800 and 2000 scales from each treatment on digital photographs processed with MotiCImages 2000 V.1.3 software [39]. Fertility was estimated based on the number of eggs produced by 50 E4 females and 50 female adults from each treatment. The females were collected, preserved individually in vials with 70% alcohol, dissected, and the number of eggs recorded. Estimating the probability of transition from egg to E1 (walker) in the field is challenging due to the short time frame involved and the fact that it occurs beneath the female adult scale. Adopting a conservative approach, we considered transition probability from egg to E1 to be equal to that from E1 to E2. This result is consistent with another species of scale insect [44]. As *T. martinezae* is sessile from the earliest stage of its life cycle, individual immigration or emigration has no relevant effect on the scale insect population growth rate estimation.

Population growth rates were estimated using the PopBio V 2.7 package in R 4.3.1 [45]. We generated 5000 random matrices for each treatment using a Monte Carlo sampling method [46] with defined probability distributions and parameter values as calculated from the field data. Permanence and transition values were sampled from binomial distributions, while fecundities were sampled from Poisson distributions. Finally, 95 percent confidence intervals for the growth rate (λ) were obtained from the 2.5th and 97.5th percentiles of the simulated λ distribution.

## 3. Results

### 3.1. Parasitism Rates

**Figure 2:**
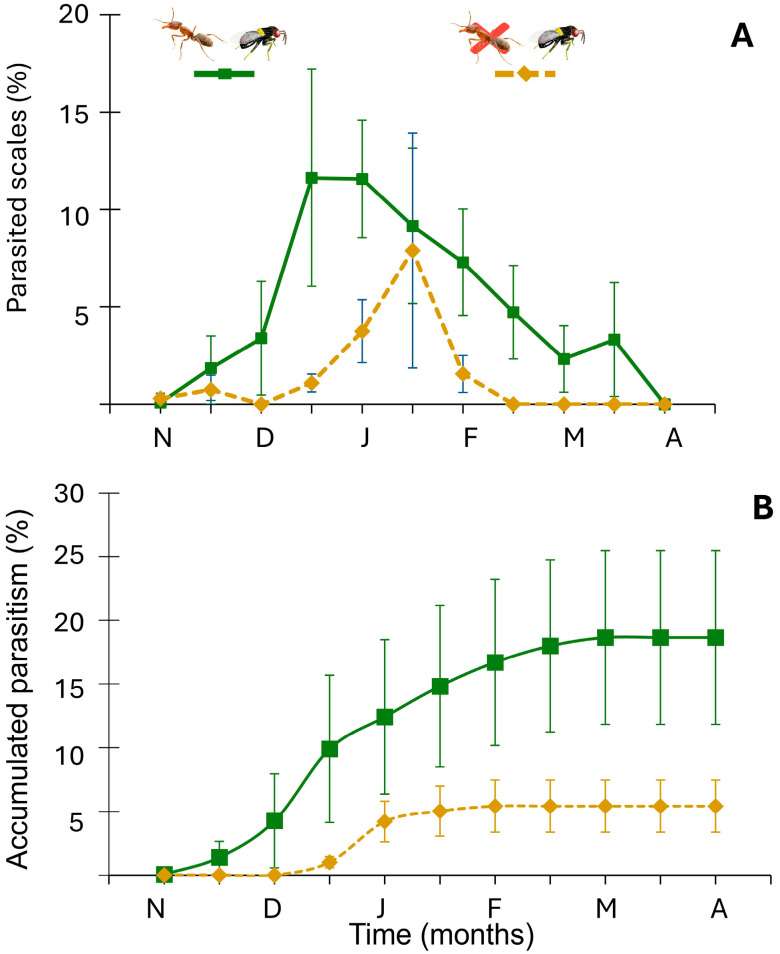
(**A**) Percentage ± SE of parasitized scales over time. (**B**) Cumulative parasitism (% ± SE ) over time. Letters denote months, from November (N) to April (A). Green solid line: presence of both the ant and the parasitoid; orange dashed line: ant excluded and presence of the parasitoid.

During the six-month monitoring period, parasitism was consistently higher in the ant-attended scale populations, despite noticeable variations between scale populations (Figure 2A). There was a significant effect of ant attendance on cumulative parasitism (X^2^_(1)_ = 5.357, *p* = 0.021). Unexpectedly, ant-attended populations averaged 18.66% parasitism (SE = 6.82%), three times the 5.42% (SE = 2.06%) average rate recorded when ants were excluded (Figure 2B). Considerable variation was observed among scale populations. For example, parasitism ranged from 1% to 52% in the ant-exclusion treatment and between 0 and 14.8% in ant-attended populations.

### 3.2. Scale Population Growth Rates

**Figure 3:**
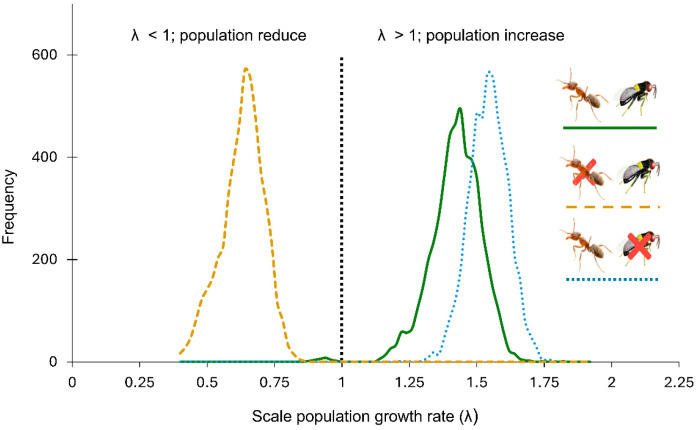
Population growth rates (λ) of *Toumeyella martinezae* under different conditions. Yellow dashed line: ant (*Liometopum apiculatum*) excluded in the presence of the parasitoid *Mexidalgus toumeyellus*; green solid line: presence of both the ant and the parasitoid; blue dashed line: ant presence in the absence of the parasitoid; orange dashed lines: ant excluded and presence of parasitoids. The *y*-axis shows the frequency distribution of λ values generated from 5000 random matrices per treatment.

Parasitoid presence reduced the growth rate of ant-attended scale populations by an estimated 8.5 percent (Table 1, Figure 3). The growth rate of parasitoid-excluded populations was estimated as λ = 1.5324 (1.3873–1.6692), compared to the λ = 1.4132 (1.2062–1.5744) in populations with parasitoid access (natural conditions). Although the presence of parasitoids reduced the scale population growth rate, the λ value remained larger than 1.0 (indicating positive growth) when ants attended the scales. In contrast, excluding ants while allowing parasitoid access had a larger impact on the scale population growth rate, driving λ down to 0.6224 (0.4600–0.7620), clearly below the replacement threshold of 1.0. This suggests that, without ant attendance, scale populations would not be demographically viable under parasitoid pressure (Figure 3).

## 4. Discussion

The parasitism rate of *Toumeyella martinezae* recorded in this study (under 18%) falls well below the 90% rate targeted by many biological control programs, and the 32% deemed necessary for successful pest control [10,14,47,48,49]. However, the rate observed in our study is comparable with the levels recorded in wild systems where ants naturally regulate pests [50,51].

Additional challenges faced by parasitoids in locating and infesting hosts in diverse natural communities—compared to monospecific crops—were highlighted by Godfray [6]. Oviposition decisions of parasitoids depend on several chemical signals, which can originate from the plant itself or be part of interactions within the system (e.g., between the plant and herbivore, the herbivore and its mutualist, or another species, i.e., a competitor) [52]. In this context, interactions with competitors, hyperparasitoids and mutualists; the low density and random spatial distribution of host plants [53]; and the mixing and dilution of signals caused by plant and herbivore diversity reduce the efficiency of the parasitoid in finding hosts.

In our study system, *Myrtillocactus geometrizans* occurred at low densities (~37 individuals ha-1) and fewer than 50% of those plants were infested by scale insects [54]. Consequently, the average distance between infested plants usually exceeded 20 m, posing a substantial obstacle for parasitoid dispersal and host detection. Moreover, *M. geometrizans* coexists with other plant species and various phytophagous insects, creating a chemically complex matrix through which parasitoids must navigate to detect and locate scale insects [55].

*T. martinezae* rarely occupies garambullo host plants in isolation. Instead, it coexists—and competes—with other sap-feeding species, including the scale *Opuntiaspis philoccocus* [56,57] and hemipterans in the genera *Pseudococcus* and *Aphis*. Chemicals released by these non-host species can mislead the parasitoid [58].

Variations in parasitism rate can be explained by the scale life cycle. Martínez-Hernández [36] recorded high parasitism rates when scales were in instars E2 and E3, and rates fell close to zero as the scales reached maturity (E4 or adulthood). This pattern was also observed in our system, suggesting that there is a brief, roughly 1.5-month window during which scales are available in high abundance in the population. This temporal dynamic is relatively common [59,60] and may explain the variability in parasitism rates observed among scale populations, due to the difficulty the parasitoid faces in locating them across a heterogeneous environment where scale-bearing plants occur in low density. Since *T. martinezae* is a bivoltine species, the parasitism rate and temporal dynamics observed may differ during the warm cycle (May–October). Including the two annual cycles of scales would provide a more complete understanding of the parasitism dynamics in this system.

Contrary to predictions based on the ant defense hypothesis, that wasps avoid ant- attended sites [61], we recorded higher parasitism in ant-attended than in ant-excluded populations. Previous studies where similar results were found (e.g., [51]) suggest that this could be explained by learning changes in wasps’ behavior in response to encounters with ants [62,63,64]. Alternatively, it could be that wasps exploit chemical cues between ants and scales linked to honeydew production [14,65,66] to locate suitable hosts and make the decision to oviposit [27]. Whether this pattern is driven by ant-scale chemical communication that attracts parasitoids, behavioral adaptations in wasps, or the greater benefit of accessing and parasitizing higher-quality scales for their offspring in relation to the potential cost of being attacked by tending-scale ants remains an open question. Clarifying this would help elucidate the indirect plant defense mechanism.

The presence of ants and the care they provide to the scales promotes the growth of larger, higher-quality scales that produce more honeydew [67,68,69,70], making scale populations more attractive to parasitoids and resulting in higher parasitism rates. *M. toumeyellus* is a tiny parasitoid that has evolved effective strategies to evade ants. It perches on the cactus spines and, as soon as the ants stop attending the scales, it quickly descends to find unparasitized scales and attack them. In other systems, it has already been reported that parasitoids modify their behavior in different ways to avoid ants or hemipteran honeydew producers that are defended by ants [27,71]. As parasitized scales continue to excrete honeydew for some time, the ants’ incentive to protect the scales from parasitoids is reduced as their resources remain largely unaffected.

Evaluating biological control effectiveness requires demographic analyses beyond [14] the parasitism rate observed at any stage of the pest’s development. In our population models, both parasitoids and ants influenced the scale population growth rate, but to a different degree. Parasitoids reduced the scale population growth rate by only 8.5%, whereas ant exclusion resulted in negative population growth (λ = 0.6224).

In our study system, the presence of the mutualistic ant modified the parasitoid effect on the scale population. In the absence of ants, parasitoid pressure would render scale populations demographically unviable, as they showed negative population growth rates. With ants present, however, the improved condition of scales [72] and the reduction in competitor pressure [56] allowed the scales to sustain positive population growth rates, despite the elevated parasitism. Removing the ants would be an effective strategy for controlling the scale pest, but this is disadvantageous due to the economic value of the ‘escamoles’ harvested from the mutualistic ant.

Managing this pest requires striking a balance between the positive effects of ants on the ecosystem and the need to keep damage and infestation levels in garambullo populations low enough to ensure their local viability. At the same time, it is important to maintain sustainable escamole extraction activities, which provide economic benefits to local communities.

The parasitoid *M. toumeyellus* thus seems to be insufficient as a standalone biological control agent for this cactus pest. Similar examples of parasitoids able to reduce the growth of phytophagous populations but insufficiently for acting as effective biological control agents have been identified. These include the parasitoid *Aphelinus certus* against the soybean aphid *Aphis glycines* [73]. Controlling this pest requires integrated pest management strategies that include managing ant mutualists to achieve suppression.

The decision of ants to attend honeydew-producing insects depends on the quantity and quality of the honeydew produced [30,74]. *L. apiculatum* acts as a facultative mutualist in the study area. In addition to attending scales, it consumes sugar-rich substances from various sources, including extrafloral nectaries of various *Opuntia* species and honeydew produced by other Hemiptera species such as *Psedococcus* sp. (mealybug) and *Dysmicoccus brevipes* (scale of *Agave salmiana*), among others. The decision of *L. apiculatum* to attend the soft-scale *T. martinezae* might depend on the reward offered and the availability of alternative sugar-rich resources in the environment. When more attractive food sources are available, ant attendance on scales may decrease, potentially leading to an increased impact of the parasitoid that may suffice to effectively control the scale pest. Providing supplemental feeding to reduce the ants’ attendance to honeydew-producing insect pests has been evaluated with promising results [75]. This strategy would need to be tested in field conditions, but could keep the ant colonies productive and reduce the negative effect of the plague over the garambullos.

In summary, our findings indicate that parasitoids’ impact as a biological control for the scale population is inadequate. Therefore, to increase the effectiveness of the parasitoid, it is necessary to reduce the protective function of the ant towards the scale.

Assessing the effectiveness of parasitoids as biological control agents in natural systems demands an integrated approach. In addition to basic information on pest population dynamics, the availability of resources and the interactions of the scale with other species must be considered. Fluctuations in resource availability commonly observed in arid environments must also be incorporated into the evaluation.

## Figures and Tables

**Table 1:** Population transition matrices for *Toumeyella martinezae* in three experimental treatments: control (ants and parasitoids allowed); with ants allowed, but parasitoids excluded; and ants excluded, but parasitoids allowed. The first row shows fertility at each life-stage class. The values of population growth rates (λ) and 95% confidence intervals are given for each matrix.

**With Ants and Parasitoids**				**λ = 1.4132 (1.2062–1.5744)**		
	Egg	E1	E2	E3	E4	Adult
Egg	0	0	0	0	69.75	371.82
E1	0.7111	0.0078	0	0	0	0
E2	0	0.7111	0.1944	0	0	0
E3	0	0	0.3451	0.5350	0	0
E4	0	0	0	0.0498	0.6010	0
Adult	0	0	0	0	0.2020	0.9200
**With Ants, Parasitoids Excluded**				**λ = 1.5324 (1.3873–1.6692)**		
	Egg	E1	E2	E3	E4	Adult
Egg	0	0	0	0	56.33	585.85
E1	0.5179	0	0	0	0	0
E2	0	0.5179	0.1877	0	0	0
E3	0	0	0.4110	0.5057	0	0
E4	0	0	0	0.1009	0.7152	0
Adult	0	0	0	0	0.1840	0.9876
**Ant Excluded, Parasitoid Allowed**				**λ = 0.6224 (0.4600–0.7620)**		
	Egg	E1	E2	E3	E4	Adult
Egg	0	0	0	0	69.75	371.82
E1	0.7784	0.0227	0	0	0	0
E2	0	0.7784	0.2533	0	0	0
E3	0	0	0.3652	0.5136	0	0
E4	0	0	0	0.0267	0.2026	0
Adult	0	0	0	0	0.0100	0.0100

## Data Availability

The raw data supporting the conclusions of this article will be made available by the authors on request.

## References

[1] Eggleton P., Belshaw R. (1992). Insect parasitoids: An evolutionary overview. Phil. Trans. R. Soc. Lond. B.

[2] Chellappan M.F., Ranjith M.T., Omkar (2023). Insect Parasitoids. Parasitoids in Pest Management.

[3] Cingolani M.F., Barakat M.C., Cerretti P., Chirinos D.T., Ferrer F., Vega J.G., Grenier S., Kondo T., Pape T., Plowes R. (2025). Dipteran parasitoids as biocontrol agents. BioControl.

[4] Thilak K.S., Parmar D.N., Rajeshbhai M.A., Sarangi S., Reddy S.N., Guntupalli S., Aulakh G.K. (2025). Parasitoids as Biological Control Agents in Agroecosystems. Role of Beneficial Insects in Agricultural Ecosystems.

[5] Eggleton P., Gaston K.J. (1990). Parasitoid species and assemblages: Convenient definition or misleading compromises?. Oikos.

[6] Godfray H.C.J. (1994). Parasitoids: Behavioral and Evolutionary Ecology.

[7] Forbes A.A., Bagley R.K., Beer M.A., Hippee C., Widmayer H.A. (2018). Quantifying the unquantifiable: Why Hymenoptera, not Coleoptera, is the most speciose animal order. BMC Ecol..

[8] Hamback P.A., Janz N., Braga M.P. (2024). Parasitoid speciation and diversification. Curr. Opin. Insect Sci..

[9] Koul O., Dhaliwal G.S., Koul O., Dhaliwal (2003). Predators and Parasitoids: An introduction. Predators and Parasitoids.

[10] Hawkins B.A., Cornell H.V., Hochberg M.E. (1997). Predators, parasitoids, and pathogens as mortality agents in phytophagous insect populations. Ecology.

[11] Vet L.E.M., Hamerik L., Visser M.E., Wäckers F.L., Lewis E.E., Campbell J.F., Sukhdeo M.V.K. (2002). Flexibility in Host-search and Patch-use Strategies of Insect Parasitoids. The Behavioural Ecology of Parasites.

[12] Hajek A. (2004). Natural Enemies: An Introduction to Biological Control.

[13] Badii M.H., Hernández-Ortiz E., Flores A.E., Landeros J. (2004). Prey stage preference and functional response of *Euseius hibisci* to *Tetranychus urticae* (Acari: Phytoseiidae, Tetranychidae). Exp. Appl. Acarol..

[14] Paris C.I., Llusia J., Peñuelas J. (2010). Changes in monoterpene emission rates of *Quercus ilex* infested by aphids tended by native or invasive *Lasius* ant species. J. Chem. Ecol..

[15] D’Alessandro M., Brunner V., von Mérey G., Turlings T.C.J. (2009). Strong Attraction of the Parasitoid *Cotesia marginiventris* Towards Minor Volatile Compounds of Maize. J. Chem. Ecol..

[16] Wei J., Wang L., Zhu J., Zhang S., Nandi O.I., Kang L. (2007). Plants Attract Parasitic Wasps to Defend Themselves against Insect Pests by Releasing Hexenol. PLoS ONE.

[17] Riddick E.W., Cottrell T.E., Kidd K.A. (2009). Natural enemies of the Coccinellidae: Parasites, pathogens, and parasitoids. Biol. Control..

[18] MacFadyen S., Davies A.P., Zalucki M.P. (2015). Assessing the impact of arthropod natural enemies on crop pests at the field scale. Insect Sci..

[19] Majerus M., Sloggett J.J., Godeau J.F., Hemptinne J.L. (2007). Interactions between ants and aphidophagous and coccidophagous ladybirds. Popul. Ecol..

[20] Stadler B., Dixon A.F.G. (2005). Ecology and Evolution of Aphid-Ant Interactions. Annu. Rev. Ecol. Evol. Syst..

[21] Singh A., Zytynska S.E., Hanna R., Weisser W.W. (2016). Ant attendance of the cotton aphid is beneficial for okra plants: Deciphering multitrophic interactions. Agric. For. Entomol..

[22] Majer J.D., Buckley R.C. (1982). Ant-plant interactions in the Darling Botanical District of Western Australia. Ant-Plant Interactions in Australia.

[23] Bristow C.M. (1984). Differential benefits from ant attendance to two species of Homoptera on New York ironweed. J. Anim. Ecol..

[24] Buckley R. (1987). Ant-plant-homopteran interactions. Advances in Ecological Research.

[25] Bristow C.M. (1991). Are ant-aphid associations a tritrophic interaction? Oleander aphids and Argentine ants. Oecologia.

[26] Yui N., Yukari M., Makoto T., Issei O. (2020). Mutualistic ants and parasitoid communities associated with a facultative myrmecophilous lycaenid, Arhopala japonica, and the effects of ant attendance on the avoidance of parasitism. Entomol. Sci..

[27] Mouratidis A., Vacas S., Herrero J., Navarro-Llopis V., Dicke M., Tena A. (2021). Parasitic wasps avoid ant-protected hemipteran hosts via the detection of ant cuticular hydrocarbons. Proc. Biol. Sci..

[28] Eisner T., Wiemer D.F., Haynes L.W., Meinwald J. (1978). Lucibufagins: Defensive steroids from the fireflies *Photinus ignitus* and *P. marginellus* (*Coleoptera*: *Lampyridae*). Proc. Natl. Acad. Sci. USA.

[29] Maschwitz U., Schroth M., Hanel H., Pong T.Y. (1984). *Lycaenids parasiting* symbiotic plants-ant partnerships. Oecologia.

[30] Fiedler K., Maschwitz U. (1988). Functional analysis of the myrmecophilous relationships between ants (*Hymenoptera*: *Formicidae*) and lycaenids (*Lepidoptera*: *Lycaenidae*)—II. Lycaenid larvae as trophobiotic partners of ants-a quantitative approach. Oecologia.

[31] Mason R.T., Fales H.M., Eisner M., Eisner T. (1991). by a Chrysopid Larva. Naturwissenschaften.

[32] Völkl W. (1995). Behavioural and morphological adaptations of the coccinellid *Platynaspis luteorubra* for exploiting ant-attended resources. J. Insect Behav..

[33] Völkl W. (1994). The effect of ant-attendance on the foraging behaviour of the aphid parasitoid *Lysiphlebus cardui*. Oikos.

[34] Tena A., Hoddle C.D., Hoddle M.S. (2013). Competition between honeydew producers in an ant-hemipteran interaction may enhance biological control of an invasive pest. Entomol. Res..

[35] Callejas-Chavero A., Martínez-Hernández D., Flores-Martínez A., Moncada-Orellana A., Diaz-Quiñones Y., Vargas-Mendoza C.F., Núñez-Farfán J., Valverde P.L. (2020). Herbivory in cacti: Fitness effects of two herbivores, one tending ant on *Myrtillocactus geometrizans* (*Cactaceae*). Evolutionary Ecology of Plant-Herbivore Interaction.

[36] Martínez-Hernández D.G. (2015). Efecto de *Liometopum apiculatum* (*Hymenoptera*: *Formicidae*) Sobre la Tasa de Parasitoidismo de *Toumeyella martinezi* (*Hemiptera*: *Coccidae*) Asociados a *Myrtillocactus geometrizans* (*Cactaceae*) en un Matorral Xerófilo de Huichapan. Bachelor’s Thesis.

[37] Kondo T., González H. (2014). A new species of *Toumeyella* Cockerell (*Hemiptera*: *Coccidae*) on *Myrtillocactus geometrizans* (*Cactaceae*) from Mexico with a checklist of known species of *Toumeyella* in the world. Insecta Mundi.

[38] Myartseva S.N., Coronado-Blanco J.M., Lomelí-Flores J.R., Martínez-Hernández D.Y. (2014). A new genus for a new species of the family Aphelinidae (*Hymenoptera*: *Chalcidoidea*) from Mexico. Zoosyst. Ross..

[39] (2002). MotiCImages 2000.

[40] Dean C.B., Lundy E.R., Balakrishnan N., Colton T., Everitt B., Piegorsch W., Ruggeri F., Teugels J.L. (2016). Overdispersion. Wiley StatsRef: Statistics Reference Online.

[41] Salinas-Ruíz J., Montesinos López O.A., Hernández Ramírez G., Crossa Hiriart J. (2023). Generalized Linear Mixed Models for Non-normal Responses. Generalized Linear Mixed Models with Applications in Agriculture and Biology.

[42] JASP Team (2025). JASP.

[43] Caswell H. (2000). Matrix Population Models: Construction, Analysis and Interpretation.

[44] Nuñez R., Ripa R., Larral P. (2008). Plagas de paltos y cítricos en Perú. Manejo de Plagas en Paltos y Cítricos.

[45] R Core Team (2020). R: A Language and Environment for Statistical Computing.

[46] Pflaumer P. (1988). Confidence intervals for population projections based on Monte Carlo methods. Int. J. Forecast..

[47] Gurr G.M., Wratten S.D. (1999). Integrated biological control: A proposal for enhancing success in biological control. Int. J. Pest Manag..

[48] Lill J.T., Marquis R.J., Ricklefs R.E. (2002). Host plants influence parasitism of forest caterpillars. Nature.

[49] Hance T., van Baaren J., Vernon P., Boivin G. (2007). Impact of extreme temperatures on parasitoids in a climate change perspective. Annu. Rev. Entomol..

[50] Itioka T., Inoue T. (1996). The consequences of ant-attendance to the biological control of the red wax scale insect *Ceroplastes rubens* by *Anicetus beneficus*. J. Appl. Ecol..

[51] Sadeghi-Namaghi H., Amiri-Jami A. (2018). Success of aphid parasitoids and their hosts varies with ant attendance: A field study. Entomol. Sci..

[52] Rodriguez-Saona C., Urbaneja-Bernat P., Salamanca J., Garzón-Tovar V. (2020). Interactive effects of an herbivore-induced plant volatile and color on an insect community in Cranberry. Insects.

[53] Fei M., Gols R., Harvey J.A. (2023). The biology and ecology of parasitoid wasps of predatory arthropods. Annu. Rev. Entomol..

[54] Suarez-Villavicencio H. (2024). Análisis de la Distribución Espacial de *Toumeyella martinezae* (*Coccidae*) en una Población de *Myrtillocactus geometrizans* (*Cactacea*) en un Matorral Xerófilo de Hidalgo. Bachelor’s Thesis.

[55] Vet L., Godfray C. (2008). Multitrophic interactions and parasitoid behavioural ecology. Behavioral Ecology of Insect Parasitoids: From Theoretical Approaches to Field Applications.

[56] Moncada-Orellana A. (2019). Competencia Entre *Toumeyella martinezae* (*Coccidae*) y *Opuntiaspis philococcus* (*Diaspididae*) Asociadas a *Myrtillocactus geometrizans* (*Cactaceae*) en Presencia y Ausencia de la Hormiga *Liometopum apiculatum* (*Formicidae*). Master’s Thesis.

[57] Moncada-Orellana A.J., Callejas-Chavero A., Sánchez-Colón S., Vargas-Mendoza C.F. (2025). Interspecific competition for space between two hemipteran phytophagous insects associated with *Myrtillocactus geometrizans* (*garambullo*) and its consequences for their population structure. Entomologia Experimentalis et Applicata.

[58] Callejas-Chavero A., Martínez-Hernández D.G., Vargas-Mendoza C.F., Flores-Martínez A. (2023). Herbivory in Myrtillocactus geometrizans (*Cactaceae*): Do parasitoids provide indirect defense or a direct advantage?. Plants.

[59] Menalled F.D., Costamagna A.C., Marino P.C., Landis D.A. (2002). Temporal variation in the response of parasitoids to agricultural landscape structure. Agric. Ecosyst. Environ..

[60] Kaneko S. (2005). Seasonal population changes of five parasitoids attacking the scale insect *Nipponaclerda biwakoensis* on the common reed, with special reference to predation by wintering birds. Entomol. Sci..

[61] Xu T., Chen L. (2021). Chemical communication in ant-hemipteran mutualism: Potential implications for ant invasions. Curr. Opin. Insect Sci..

[62] Barzman M.S., Daane K.M. (2001). Host-handling behaviours in parasitoids of the black scale: A case for ant-mediated evolution. J. Anim. Ecol..

[63] Völkl W. (2001). Parasitoid learning during interactions with ants: How to deal with an aggressive antagonist. Behav. Ecol. Sociobiol..

[64] Kaneko S. (2003). Different Impacts of Two Species of Aphid-attending Ants with Different Aggressiveness on the Number of Emerging Adults of the Aphid’s Primary Parasitoid and Hyperparasitoids. Ecol. Res..

[65] Orr M.R., De Camargo R.X., Benson W.W. (2003). Interactions between ant species increase arrival rates of an ant parasitoid. Anim. Behav..

[66] Heil M. (2008). Indirect defence via tritrophic interactions. New Phytol..

[67] Billick I., Hammer S., Reithel J.S., Abbot P. (2007). Ant–aphid interactions: Are ants’ friends, enemies, or both?. Ann. Entomol. Soc. Am..

[68] Nowak H., Komor E. (2010). How aphids decide what is good for them: Experiments to test aphid feeding behaviour on *Tanacetum vulgare* (L.) using different nitrogen regimes. Oecologia.

[69] Tena A., Wäckers F.L., Heimpel G.E., Urbaneja A., y Pekas A. (2016). Parasitoid Nutritional Ecology in a Community Context: The Importance of Honeydew and Implications for Biological Control. Curr. Opin. Insect Sci..

[70] Migani V., Ekesi S., Merkel K., Hoffmeister T. (2017). At lunch with a killer: The effect of weaver ants on host-parasitoid interactions on mango. PLoS ONE.

[71] Dicke M., Grostal P. (2001). Chemical detection of natural enemies by arthropods: An ecological perspective. Annu. Rev. Ecol. Syst..

[72] González Villa H. (2021). Efecto del Parasitoidismo y Mutualismo Sobre la Demografía de *Toumeyella martinezae* (*Hemíptera*: *Coccidae*) Asociada a *Myrtillocactus geometrizans* (*Cactaceae*) en un Matorral Xerófilo de Huichapan, Hidalgo. Masters’ Thesis.

[73] Miksanek J.R., Heimpel G.E. (2019). A matrix model describing host–parasitoid population dynamics: The case of *Aphelinus certus* and soybean aphid. PLoS ONE.

[74] Nelson A.S., Mooney K.A. (2022). The evolution and ecology of interactions between ants and honeydew-producing hemipteran insects. Annu. Rev. Ecol. Evol. Syst..

[75] Burgio G., Magagnoli S., Casoli L., Profeta M., Grasso D.A., Schifani E., Giannetti D., Parrilli M. (2025). Use of Sugar Dispensers at Lower Density Can Decrease Mealybug (*Hemiptera*: *Pseudococcidae*) Infestation in Vineyards by Disrupting Ants. Insects.

